# Neuroinvasive virus facilitates viral replication by employing lipid droplets to reduce arachidonic acid-induced ferroptosis

**DOI:** 10.1016/j.jbc.2024.107168

**Published:** 2024-03-13

**Authors:** Jianqing Zhao, Qianruo Wang, Zhenkun Liu, Mai Zhang, Jinquan Li, Zhen F. Fu, Ling Zhao, Ming Zhou

**Affiliations:** 1 National Key Laboratory of Agricultural Microbiology, Huazhong Agricultural University, Wuhan, China; 2 Key Laboratory of Preventive Veterinary Medicine of Hubei Province, College of Veterinary Medicine, Huazhong Agricultural University, Wuhan, China; 3 Frontiers Science Center for Animal Breeding and Sustainable Production, Wuhan, China; 4 College of Biomedicine and Health, College of Life Science and Technology, Huazhong Agricultural University Wuhan, China; 5 Hubei Hongshan Laboratory, Wuhan, China

**Keywords:** neuroinvasive virus, lipid droplets, diacylglycerol acyltransferase, arachidonic acid, lipid peroxidation, ferroptosis

## Abstract

Lipids have been previously implicated in the lifecycle of neuroinvasive viruses. However, the role of lipids in programmed cell death and the relationship between programmed cell death and lipid droplets (LDs) in neuroinvasive virus infection remains unclear. Here, we found that the infection of neuroinvasive virus, such as rabies virus and encephalomyocarditis virus could enhance the LD formation in N2a cells, and decreasing LDs production by targeting diacylglycerol acyltransferase could suppress viral replication. The lipidomics analysis revealed that arachidonic acid (AA) was significantly increased after reducing LD formation by restricting diacylglycerol acyltransferase, and AA was further demonstrated to induce ferroptosis to inhibit neuroinvasive virus replication. Moreover, lipid peroxidation and viral replication inhibition could be significantly alleviated by a ferroptosis inhibitor, ferrostatin-1, indicating that AA affected neuroinvasive virus replication mainly through inducing ferroptosis. Furthermore, AA was demonstrated to activate the acyl-CoA synthetase long-chain family member 4-lysophosphatidylcholine acyltransferase 3-cytochrome P450 oxidoreductase axis to induce ferroptosis. Our findings highlight novel cross-talks among viral infection, LDs, and ferroptosis for the first time, providing a potential target for antiviral drug development.

Neuroinvasive viruses are a series of viruses, including DNA viruses and RNA viruses, that can penetrate the blood-brain barrier to mainly infect central nervous system, such as rabies virus (RABV), severe acute respiratory syndrome coronavirus 2, poliovirus, and encephalomyocarditis virus (EMCV), and so on. Neuroinvasive viruses can cause fatal diseases, which pose a threat to global public health (1, 2). Intriguingly, lipid substances have been demonstrated to act as an important role in neuroinvasive viruses production, indicating the significance of lipid metabolism in virus lifecycle (3). Lipid has been linked to central neurological damage in neuroinvasive virus infection in previous studies (4, 5). Hence, understanding cellular lipid metabolism is critical for revealing the pathogenesis of neuroinvasive virus.

As the main place to store cellular neutral lipids, lipid droplets (LDs) mainly store triglycerides and cholesterol esters (6). Besides the triglycerides and cholesterol esters in LDs, several fatty acids (FAs), especially long-chain polyunsaturated acids (PUFAs), are also reported to exist in LDs (7). FAs, including PUFAs, are the main components of triacylglycerol (TAG) which are formed by monoacylglycerol and diacylglycerol (DAG) *via* a series of enzymes. DAG is catalyzed by diacylglycerol acyltransferase 1/2 (DGAT1/2) to form TAG, which is the main rate-limiting reaction in this process (8). DGAT1/2 enzyme has been demonstrated to suppress RABV production by restricting viral budding in our previous study (9). However, whether the lipid metabolism regulated by DGAT can affect other neuroinvasive virus infection is still unclear.

PUFAs generally consist of omega-3 (n-3) and omega-6 (n-6) (10). Arachidonic acid (AA), which belongs to n-6 PUFAs, is usually esterified into membrane phospholipids (PL) that are widely distributed in mammalian cells (11), and it is a tetra-unsaturated FA (*cis*-5,8,11,14) that acts as a critical role in cellular membrane fluidity, enzymatic transformations, and cell proliferation (12). As the direct precursor of bioactive lipid roles such as prostaglandin, leukotrienes, epoxyeicosatrienoic acids, and endocannabinoids (13). AA is mainly sourced from two ways: (1) hydrolysis of membrane PL mediated by phospholipase A2 (14); (2) synthesis *via* linolenic acid (LA)-γ-Linolenic acid (GLA)-dihomo-γ-linolenic acid (DGLA)-AA route (15, 16). Although AA has been investigated in nutrition (17), infant development (18), cancer treatment (19), inflammation (20, 21), ferroptosis (22, 23), and viral infection (24), the exact role of AA in neuroinvasive virus infection remains unclear.

A previous study has demonstrated that AA is the main substrate of lipid peroxidation in ferroptosis (23), and ferroptosis is a kind of programmed cell death (PCD) that has been noticed recently (25, 26). PCD has been demonstrated to affect viral inhibition (27, 28). It has been reported that ferroptosis could inhibit infection of several viruses, including newcastle disease virus (29), simian immunodeficiency virus (30) and hepatitis C virus (31). However, some viruses such as enterovirus and coronavirus, could induce ferroptosis to facilitate viral replication, indicating that ferroptosis may play different roles in different viruses (32). For neuroinvasive viruses, the role of cellular lipid metabolism in viral replication is still unclear.

In the present study, we found that neuroinvasive virus infection could enhance the LD formation while targeting DGAT1/2 to reduce LD production could suppress neuroinvasive virus infection by increasing the release of cellular AA. The increased cellular AA could further induce lipid peroxidation and ferroptosis to restrict neuroinvasive virus replication. Our results reveal a novel interplay among LDs, ferroptosis, and neuroinvasive virus infection, providing a promising target for antiviral drug development for the neuroinvasive virus.

## Results

### Decreasing LD formation by reducing TAG biogenesis with DGAT inhibitors suppresses infection of RABV or EMCV

**Figure 1:**
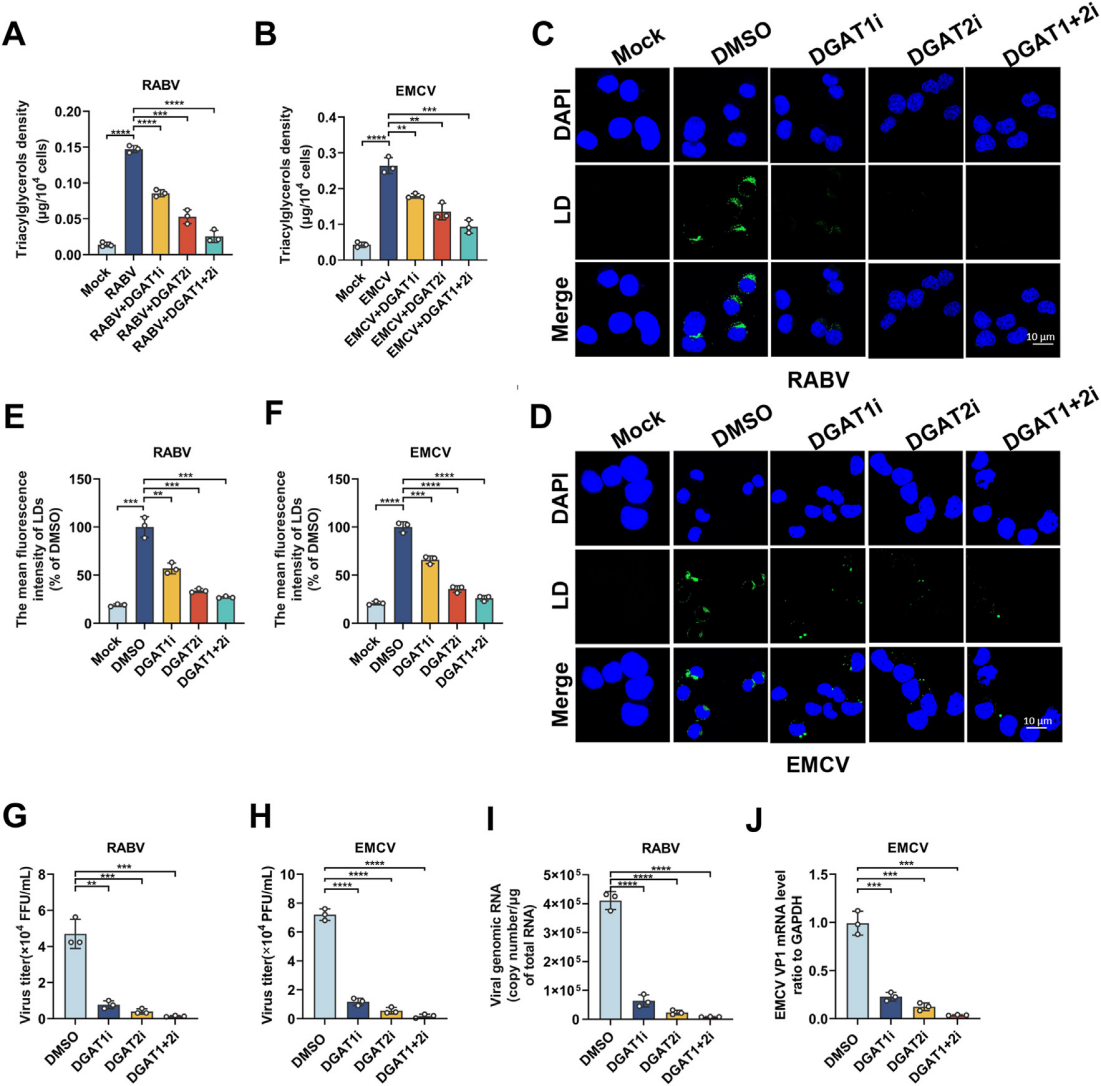
**Targeting DGAT to inhibit TAG biogenesis and LD formation suppresses neuroinvasive virus production.** N2a cells were pretreated with DGAT1i (A922500, 10 μg/ml), DGAT2i (PF06424439, 60 μg/ml) DGAT1+2i (A922500, 10 μg/ml + PF06424439, 60 μg/ml) or DMSO, and infected with RABV (MOI = 0.1, 24 h) (*A*) or EMCV (MOI = 1, 24 h) (*B*), cellular triacylglycerols (TAG) were tested by ELISA kit (n = 3). N2a cells were pretreated with DGAT1i (A922500, 10 μg/ml), DGAT2i (PF06424439, 60 μg/ml) DGAT1+2i (A922500, 10 μg/ml + PF06424439, 60 μg/ml) or DMSO, and infected with RABV (MOI = 0.1, 24 h) (*C*) or EMCV (MOI = 1, 24 h) (*D*), lipid droplets (LDs) were stained by BODIPY493/503, and the mean fluorescence of LDs (*E* and *F*) were calculated individually (n = 3). N2a cells were pretreated with DGAT1i, DGAT2i, or DGAT1+2i and infected with RABV (MOI = 0.1, 24 h) (*G*), and EMCV (MOI = 1, 24 h) (*H*) supernatants were harvested and determined virus titers (n = 3). Cells were collected to test RABV genome RNA (*I*) and EMCV-VP1 mRNA (*J*) by qPCR (n = 3). Error bars represent mean ± SD. Statistical analysis was determined by one-way ANOVA or Student's *t* test and notated as follows: ∗*p* < 0.05; ∗∗*p* < 0.01; ∗∗∗*p* < 0.001 and ∗∗∗∗*p* < 0.0001. The scale bar represents 10 μm. DGAT, diacylglycerol acyltransferase; DMSO, dimethyl sulfoxide; EMCV, encephalomyocarditis virus; qPCR, quantitative PCR; RABV, rabies virus.

Our previous study has demonstrated that LDs play an important role in RABV replication, also revealed the critical role of DGAT which catalyzes TAG synthesis from DAG in LD formation (9). However, whether DGAT plays an important role in neuroinvasive virus infection is still unclear. Hence, in this study, the mouse neuroblastoma cell line N2a was treated with DGAT inhibitors DGAT1i (A922500, 10 μg/ml), DGAT2i (PF06424439, 60 μg/ml) or DGAT1i + DGAT2i (A922500+PF06424439, 10 μg/ml+60 μg/ml), and then infected with RABV or EMCV. Cellular TAG, LDs, viral titer, and viral RNA level were determined after DGAT inhibitors treatment individually. As shown in Figure 1, *A* and *B*, the levels of TAG after DGAT inhibitors treatment were significantly decreased in RABV or EMCV infected cells, respectively. Correspondingly, the numbers of LDs were significantly reduced along with the decrease of TAG in RABV and EMCV infection as shown in Figure 1, *C* and *D*, the mean fluorescence intensity of LDs were calculated, respectively (Fig. 1, *E* and *F*). Moreover, the viral titers (Fig. 1, *G* and *H*) and viral RNA levels (Fig. 1 , *I*and *J*) of RABV, and EMCV were significantly decreased in infected cells after DGAT inhibitors treatment, respectively.

**Figure 2:**
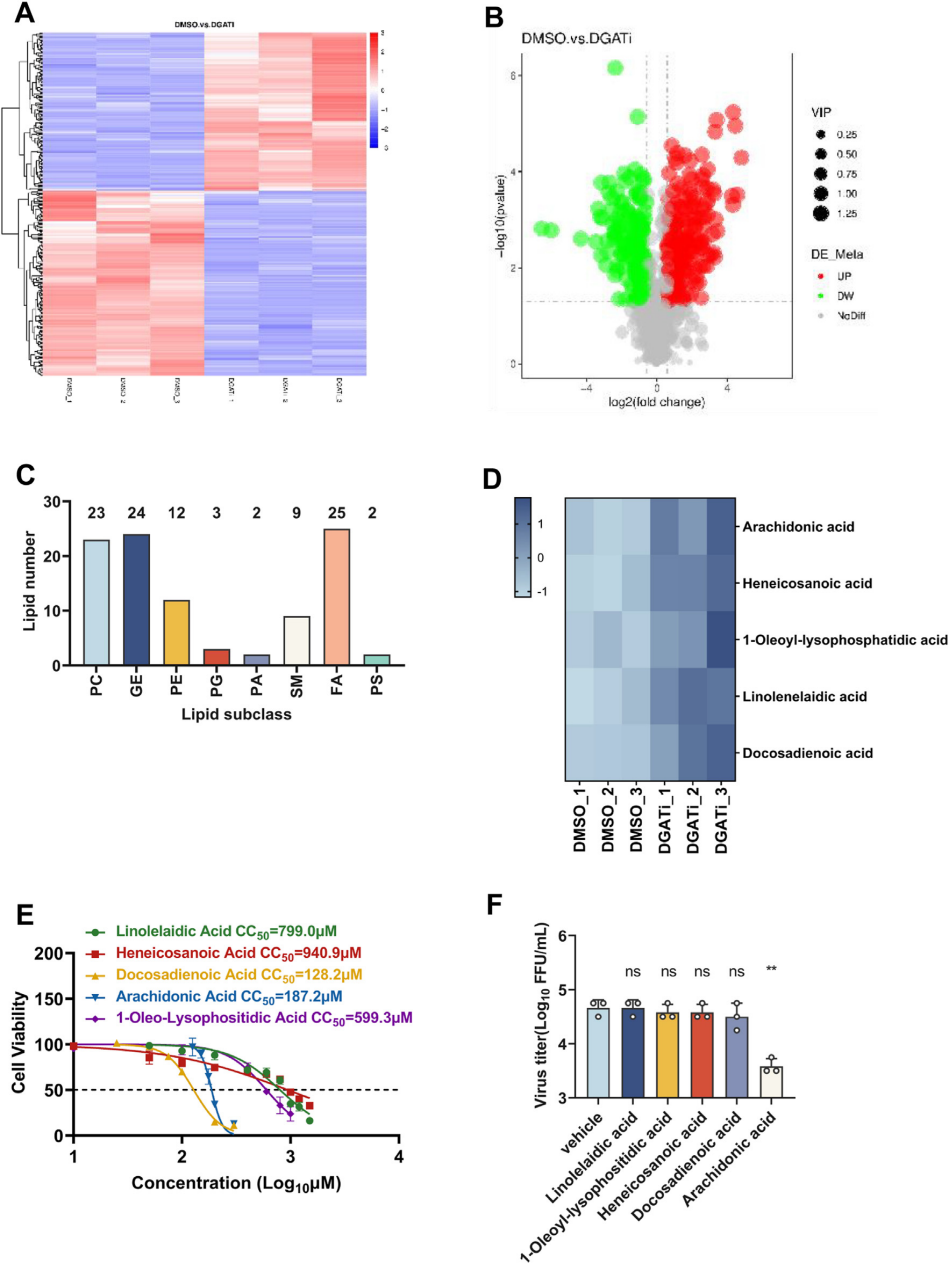
**Lipidomics analysis of DGAT-inhibiting groups and DMSO groups.***A*, heatmap of differentially expressed fatty acids. Each row is corrected for the Z value. Longitudinal is the clustering of samples, and horizontal is the clustering of lipid substances. *B*, volcano plot of lipid substances. *Gray* plots represent the lipid substance's insignificant differences, *red* plots represent the lipid substances upregulated, and *green* plots represent the lipid substances downregulated. *C*, partially categories of lipid substances are classified. *D*, heatmap of five increased fatty acids (arachidonic acid, heneicosanoic acid, 1-oleoyl-lysophosphatidic acid, linolenelaidic acid,and docosadienoic acid). *E*, N2a cells were treated with lipid substances including arachidonic acid, heneicosanoic acid, 1-oleoyl-lysophosphatidic acid, linolenelaidic acid,and docosadienoic acid with gradient concentrations for 48 h. Cell viability was determined with cell counting kit 8 (CCK-8) and cytotoxicity 50% (CC_50_) of lipid substances were calculated respectively (n = 3). *F*, N2a cells were treated with lipid substances (AA, 100 μm; heneicosanoic acid, 15 μm; 1-oleoyl-lysophosphatidic acid, 20 μm; linolenelaidic acid, 50 μm; docosadienoic acid, 50 μm) for 24 h, supernatants were harvested and determined virus titers (n = 3). Statistical analysis was determined by Student's *t* test and notated as follows: ∗∗*p* < 0.01 and ns, no significant. AA, arachidonic acid; DGAT, diacylglycerol acyltransferase; DMSO, dimethyl sulfoxide.

Decreasing LD formation through affecting TAG synthesis induces the accumulation of AA to inhibit RABV infection. We have demonstrated that inhibiting DGAT restricts neuroinvasive virus infection by interfering with LD production. Due to the important role of DGAT in cellular lipid metabolism, the change in cellular lipid metabolism in DGAT inhibition was further explored. Therefore, to comprehensively reveal the role of DGAT in neuroinvasive virus infection, a lipidomics analysis was carried out in RABV infected N2a cells as a model for neuroinvasive virus infection. Briefly, N2a cells were treated with DGATi (A922500+PF06424439) or dimethyl sulfoxide (DMSO), and then infected with RABV (multiplicity of infection (MOI) = 0.1, 24 h), the changes of lipid composition in infected cells were assessed by the lipidomics analysis. All lipid components were displayed in the form of cluster heatmap (Fig. 2*A*), indicating that there are 150 types of lipid substances increasing and 175 types of lipid substances decreasing after DGATi treatment *via* a volcano plot (Fig. 2*B*). Due to the inhibition of TAG biosynthesis could directly increase cellular monoacylglycerol, DAG and FAs, we therefore focused on the increase of lipid substances. Based on the analysis of LIPID MAPS and Lipid Blast database, only 100 types of lipid substances were classified clearly including 25 species of FAs, 24 types of glycerides, 23 species of phosphatidylcholine, 12 species of phosphatidylethanolamine (PE), 9 species of sphingomyelin, 3 species of phosphatidylglycerol, 2 species of phosphatidic acid and 2 species of phosphatidylserine as shown in Figure 2*C*. FA has been demonstrated to regulate viral replication and production of many viruses (31, 33), and it was also found to occupy the main types of lipid substances. Hence, whether these upregulated FA could affect neuroinvasive virus production was further determined.

Among these 25 types of FAs, the 5 highly ranked types of FAs were available in our laboratory including AA, heneicosanoic acid, 1-oleoyl-lysophosphatidic acid, linolenelaidic acid, and docosadienoic acid (Fig. 2*D*).To further characterize the role of these upregulated FA in neuroinvasive viral infection, the viral titers of RABV were determined after the treatment of the selected FA. Initially, the concentration of cytotoxicity 50% (CC_50_) of indicated FA in N2a cells were evaluated by cell counting kit 8 as shown in Figure 2*E*, indicating that CC_50_ of AA, heneicosanoic acid, 1-oleoyl-lysophosphatidic acid, linolenelaidic acid, and docosadienoic acid were 187.2 μM, 940.9 μM, 599.3 μM, 799.0 μM, and 128.2 μM, respectively. Then, based on the CC_50_ curves of these FA, N2a cells were pretreated with these FAs at no cytotoxicity concentrations (AA, 100 μm; heneicosanoic acid, 15 μm; 1-oleoyl-lysophosphatidic acid, 20 μm; linolenelaidic acid, 50 μm; docosadienoic acid, 50 μm), and the above concentration of each FA would be no significant cytotoxic in N2a cells based on the fit curves. Then the cells were infected with RABV (MOI = 0.1) for 24 h. As shown in Figure 2*F*, only AA treatment could inhibit RABV infection with a decrease of approximately 1 log virus titer.

### Arachidonic acid restricts neuroinvasive virus replication stage of viral lifecycle

**Figure 3:**
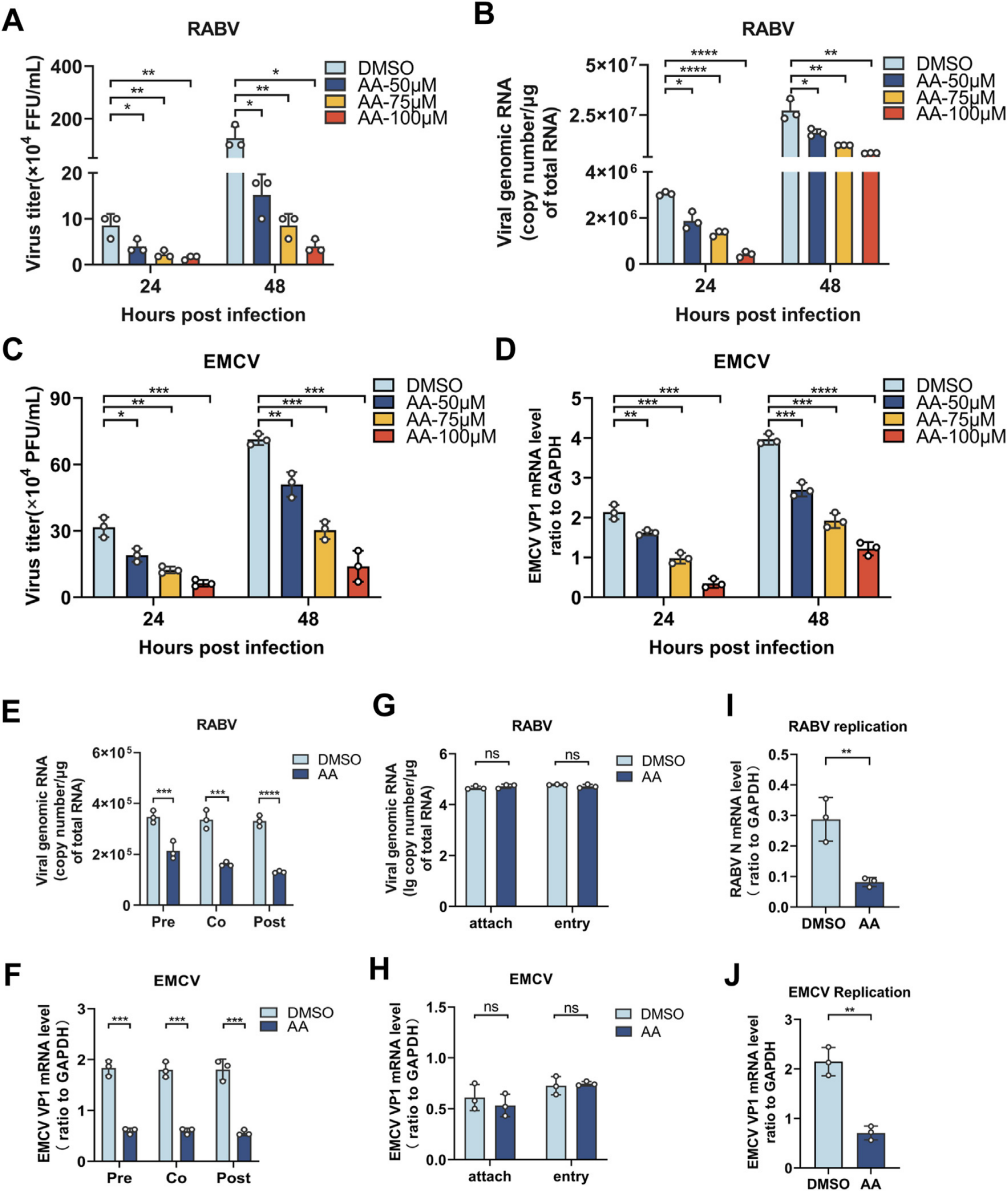
**Arachidonic Acid restricts neuroinvasive virus production.** N2a cells were treated with arachidonic acid (0 μM, 50 μM, 75 μM, and 100 μM) and infected with RABV (MOI = 0.1) for 24 and 48 h, supernatants were harvested and determined virus titers (n = 3) (*A*) and viral genome RNA was tested by qPCR (n = 3) (*B*). N2a cells were pretreated with AA in several concentrations (0 μM, 50 μM, 75 μM, and 100 μM) and infected with EMCV (MOI = 1) at the indicated time (24 h and 48 h), supernatants were harvested to determine virus titers (n = 3) (*C*) and cells were collected to test VP1 mRNA level *via* qPCR (n = 3) (*D*). N2a cells were pretreated with arachidonic acid (100 μM) for 2 h and infected with RABV (MOI = 0.1) for 24 h, or cotreated with arachidonic acid (100 μM) and infected with RABV (MOI = 0.1) for 24 h, or infected with RABV (MOI = 0.1) for 2 h and posttreated with RABV (MOI = 0.1) for 24 h, viral genome RNA were determined by qPCR (*E*). N2a cells were pre-treated with arachidonic acid (100 μM) for 2 h and infected with EMCV (MOI = 1) for 24 h, or cotreated with arachidonic acid (100 μM) and infected with EMCV (MOI = 1) for 24 h, or infected with RABV (MOI = 0.1) for 2 h and posttreated with EMCV (MOI = 1) for 24 h, VP1 mRNA were determined by qPCR (*F*). N2a cells were pretreated with arachidonic acid (100 μM) and infected with RABV (MOI = 0.1) at 4 °C for 1 h to assess viral attachment (n = 3) or at 37 °C for 2 h to assess viral internalization by qPCR (n = 3) (*G*). N2a cells were pretreated with arachidonic acid (100 μm) and infected with EMCV (MOI = 1) at 4 °C for 1 h to assess viral attachment (n = 3) or at 37 °C for 2 h to assess viral internalization by qPCR (n = 3) (*H*). N2a cells were treated with arachidonic acid (100 μM) and infected with RABV (MOI = 0.1) for 24 h, RABV N mRNA was tested by qPCR (n = 3) (*I*). N2a cells were treated with arachidonic acid (100 μM) and infected with EMCV (MOI = 1) for 24 h, VP1 mRNA was tested by qPCR (n = 3) (*J*). Error bars represent mean ± SD. Statistical analysis was determined by Student's *t* test and is notated as follows: ∗*p* < 0.05; ∗∗*p* < 0.01; ∗∗∗*p* < 0.001, ∗∗∗∗*p* < 0.0001 and ns, not significant. AA, arachidonic acid; EMCV, encephalomyocarditis virus; qPCR, quantitative PCR; RABV, rabies virus.

We have demonstrated that AA treatment could restrict RABV infection, to further investigate whether AA treatment could suppress the infection of the neuroinvasive virus, including RABV, in a dose dependant manner, N2a cells were pretreated with different concentrations (50 μM, 75 μM, and 100 μM) of AA, and then infected with RABV (MOI = 0.1), or EMCV (MOI = 1), and the supernatants and cell lysates were harvested at indicated time points (24 h and 48 h) to determine virus titers and viral RNA, respectively. The virus titers (Fig. 3, *A* and *C*) and viral RNA levels (Fig. 3, *B* and *D*) of RABV, and EMCV were significantly decreased after AA treatment in a dose-dependent manner. Therefore, 100 μM of AA treatment was used for the following experiments.

Moreover, to investigate whether the process order of viral infection and AA treatment affects virus production, the pretreatement, cotreatment, and posttreatment of AA were tested, respectively, indicating that the virus inhibition were all observed in different orders in both RABV and EMCV infection (Fig. 3, *E* and *F*). Furthermore, to determine the inhibitory stage of AA treatment on neuroinvasive virus lifecycle, the viral lifecycle was divided into three stages, including attachment, entry, and replication, and the inhibitory effects of AA treatment on different stages of viral lifecycle were assessed by measuring viral RNA levels. Briefly, N2a cells pretreated with AA were infected with RABV or EMCV at 4 °C for 1 h, and the effect of AA treatment on viral attachment was determined by quantitative PCR (qPCR). For viral entry, N2a cells pretreated with AA were infected with RABV or EMCV at 37 °C for 2 h, and the effect of AA treatment on viral entry was determined by qPCR. As shown in Figure 3, *G* and *H*, AA treatment did not affect the viral attachment and entry of RABV, and EMCV, respectively. For viral replication, N2a cells pretreated with AA were infected with RABV or EMCV at 37 °C for 24 h, and the effect of AA treatment on viral replication was determined by qPCR. As shown in Figure 3, *I* and *J*, the RABV N mRNA levels and EMCV capsid protein VP1 mRNA level were significantly decreased after AA treatment, respectively. These results indicated that AA treatment mainly affects the viral replication of neuroinvasive viruses.

Meanwhile, to investigate whether the viral inhibition of AA occurs in different MOI infection, N2a cells pretreated with AA were infected with RABV (MOI = 0.01 and 0.1) or EMCV (MOI = 0.1 and 1) at 37 °C for 24 h, virus titration, viral genome RNA, and VP1 were determined, respectively. Results indicated that AA significantly inhibited RABV production (Fig. S1*A*) and genome RNA (Fig. S1*B*) in both different infection, which is consistent in EMCV production (Fig. S1*C*) and VP1 mRNA level (Fig. S1*D*).

### Arachidonic acid inhibits neuroinvasive virus replication mainly by inducing ferroptosis

**Figure 4:**
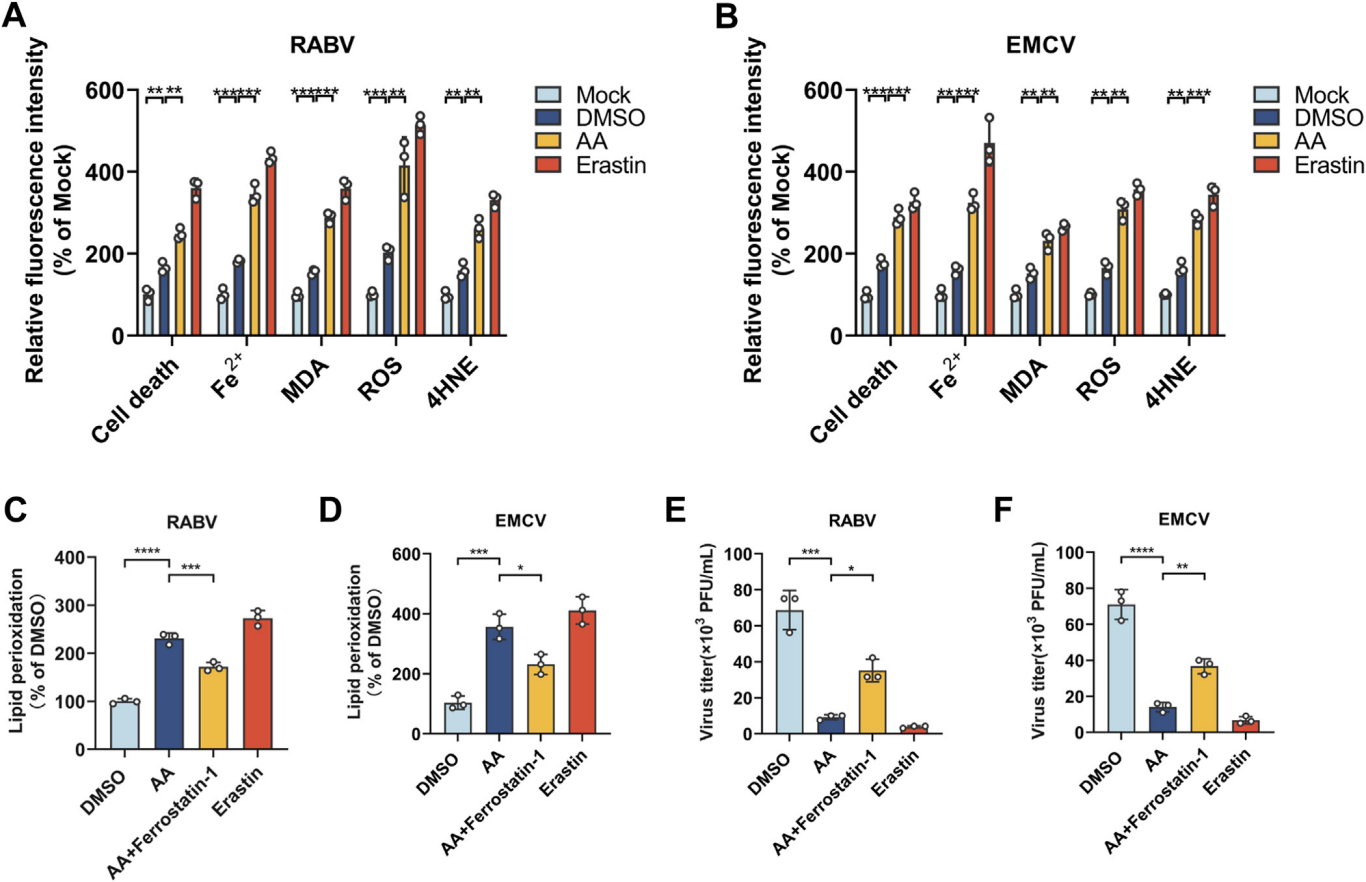
**Arachidonic acid inhibits neuroinvasive virus production by inducing ferroptosis.** N2a cells were treated with arachidonic acid (100 μM) and infected with RABV (MOI = 0.1) for 24 h, cells were harvested and stained with PI, Liperfluo, FerroOrange, DCFH-DA, or 4-HNE antibody to determine cell death, Fe^2+^, MDA, ROS, 4-HNE (n = 3) (*A*), or lipid peroxidation (n = 3) (*C*) by a fluorescent microplate reader, erastin (10 μM) was used as positive control. N2a cells were treated with arachidonic acid (100 μM) and infected with EMCV (MOI = 1) for 24 h, cells were harvested and stained with PI, Liperfluo, FerroOrange, DCFH-DA, or 4-HNE antibody to determined cell death, Fe^2+^, MDA, ROS, 4-HNE (n = 3) (*B*), or lipid peroxidation (n = 3) (*D*) by a fluorescent microplate reader, erastin (10 μM) was used as a positive control. Treated N2a cells (AA,100 μM or AA,100 μM + ferrostatin-1, 10 μM) were infected with RABV (MOI = 0.1) (n = 3) (*E*) or EMCV (MOI = 1) (n = 3) (*F*) for 24 h, supernatants were harvested to test virus titers. Error bars represent mean ± SD. Statistical analysis was determined by Student's *t* test and is notated as follows: ∗*p* < 0.05; ∗∗*p* < 0.01; ∗∗∗*p* < 0.001 and ∗∗∗∗*p* < 0.0001. 4-HNE, hydroxynonenal; AA, arachidonic acid; EMCV, encephalomyocarditis virus; MDA, malondialdehyde; PI, propidium iodide; RABV, rabies virus; ROS, reactive oxygen species.

AA has been demonstrated to act as a main component in ferroptosis (34). Hence, we hypothesized that AA could inhibit neuroinvasive virus replication by inducing ferroptosis. Hence, hydroxynonenal (4-HNE), malondialdehyde (MDA) and reactive oxygen species (ROS) were tested, respectively. Briefly, N2a cells were pretreated with AA or DMSO and infected with RABV (MOI = 0.1) or EMCV (MOI = 1), cells were collected and stained with propidium iodide (PI), FerroOrange, and 2',7'-dichlorodihydrofluorescein diacetate (DCFH-DA) to determine cell death, Fe^2+^ concentration and ROS by testing fluorescence *via* a microplate reader. Cellular 4-HNE concentration was assessed by staining with 4-HNE antibody and labeling by Alexa Fluor 488 antibody to test fluorescence read by a microplate reader. MDA concentration was determined by MDA assay kit *via* testing the fluorescence of thiobarbituric acid complex read by a microplate reader. As shown in Figure 4, *A* and *B*, significantly more cell deaths, high concentrations of Fe^2+^, MDA, 4-HNE, and ROS were all observed in neuroinvasive virus infected cells with DMSO treatment than those in uninfected (mock) cells, while significantly more cell deaths were observed in neuroinvasive virus infected cells with AA treatment than those in DMSO-treated cells.

Of note, lipid peroxidation is a specific index of ferroptosis. Hence, we further determined the cellular lipid peroxidation level in neuroinvasive virus infected N2a cells with or without AA treatment, and a ferroptosis inhibitor (ferrostatin-1, 10 μM) was also used to identify whether ferroptosis is the main response to AA treatment. As shown in Figure 4, *C* and *D*, the cellular lipid peroxidation levels in each neuroinvasive virus infected N2a cells were significantly increased compared with those in uninfected cells (mock), and the cellular lipid peroxidation levels were significantly elevated in each neuroinvasive virus infected N2a cells with AA treatment than those in cells with DMSO treatment, while the addition of ferrostatin-1 could significantly decrease the cellular lipid peroxidation levels, respectively. Moreover, the virus titer of each neuroinvasive virus was significantly recovered with the addition of ferrostatin-1 compared with those in AA-treated N2a cells without ferrostatin-1 incubation, as shown in Figure 4, *E* and *F*, suggesting that AA inhibits neuroinvasive viral replication mainly through inducing ferroptosis in N2a cells. Furthermore, the AA concentration in N2a cells infected with neuroinvasive virus were determined. As shown in Fig. S2*A*, total AA concentration was significantly increased after neuroinvasive virus infection. Moreover, LDs were extracted from infected N2a cells, the concentration of AA in purified LDs and cell lysates were determined. The results indicated that the AA concentration in LDs from infected N2a cells was significantly increased (Fig. S2*B*), while the intracellular AA concentration was significantly decreased (Fig. S2*C*), indicating that the increased LD formation after neuroinvasive virus infection could enhance the LD storage of AA to decrease the intracellular AA concentration, which would reduce the cellular AA induced ferroptosis to facilitate viral replication.

### Targeting DGAT suppresses neuroinvasive virus replication through inducing AA-dependent ferroptosis

**Figure 5:**
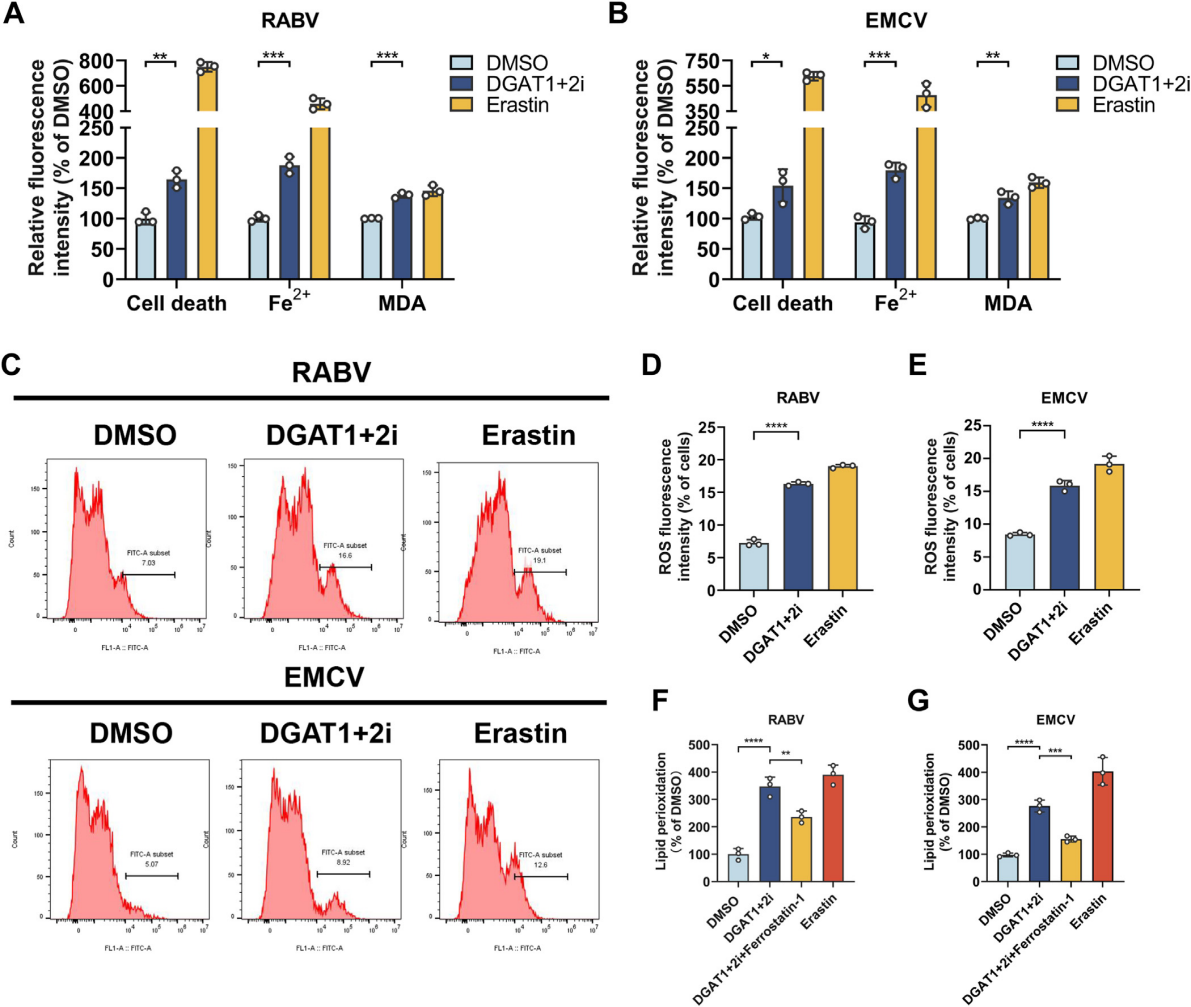
**Targeting DGAT to suppress neuroinvasive virus production through inducing arachidonic acid-dependent lipid peroxidation and ferroptosis.** Pretreated N2a cells were infected with RABV (MOI = 0.1, 24 h), cells were harvested and determined cell death, Fe^2+^, MDA (*A*), and lipid peroxidation (*F*) by a fluorescent microplate reader (n = 3). Erastin (10 μM) was used as a positive control. Pretreated N2a cells were infected with EMCV (MOI = 1, 24 h), cells were harvested and determined cell death, Fe^2+^, MDA, (*B*) and lipid peroxidation (*G*) by a fluorescent microplate reader (n = 3). Erastin (10 μM) was used as a positive control. Pretreated N2a cells (DGAT1+2i) were infected with RABV (MOI = 0.1), and EMCV (MOI = 1) for 24 h, cells were collected and determined ROS *via* flow cytometry (*C*) and calculated (*D* and *E*) (n = 3). Error bars represent mean ± SD. Statistical analysis was determined by Student's *t* test and is notated as follows: ∗*p* < 0.05; ∗∗*p* < 0.01; ∗∗∗*p* < 0.001, and ∗∗∗∗*p* < 0.0001. DGAT, diacylglycerol acyltransferase; EMCV, encephalomyocarditis virus; MDA, malondialdehyde; RABV, rabies virus; ROS, reactive oxygen species.

Although we demonstrated that AA could induce ferroptosis to inhibit neuroinvasive virus replication, whether inhibition of DGAT with inhibitors could induce AA-dependent ferroptosis to restrict neuroinvasive virus replication in neuronal cells is still unclear. Hence, N2a cells were treated with DGAT1+2i (A922500+PF06424439, 10 μg/ml+60 μg/ml), and then infected with RABV (MOI = 0.1) or EMCV (MOI = 1), and the indexes of ferroptosis that cell death percentage, Fe^2+^ concentration, and MDA level were determined and read by a fluorescent microplate reader (Fig. 5, *A* and *B*). Consistent with AA treatment, DGAT inhibitors could significantly increase the cell death percentage, the concentration of Fe^2+^, and levels of MDA in each neuroinvasive virus infected N2a cells, respectively. Meanwhile, the cellular ROS level was detected by flow cytometry, and the positive fluorescent intensity was also calculated. Indeed, significantly more ROS-positive signals were observed in cells with DGAT inhibitors addition as shown in Figure 5*C* (fluorescence-activated cell sorting analysis) and Figure 5, *D* and *E* (fluorescent intensity calculation). Furthermore, the most important index of ferroptosis, cellular lipid peroxidation level, was also determined, and DGAT inhibitors could significantly increase cellular lipid peroxidation level in each neuroinvasive virus infected N2a cells, while the addition of ferroptosis inhibitor (ferrostatin-1) could significantly reduce cellular lipid peroxidation level as shown in Figure 5, *F* and *G*. Meanwhile, to investigate whether DGAT-inhibition induces ferroptosis was due to the increase of intracellular AA, the intracellular AA concentration in N2a cells with DGAT inhibition treatment or with exogenous AA treatment in the context of neuroinvasive virus infection was also determined. As shown in Fig. S2*D*, no significant difference on the concentration of intracellular AA was observed between exogenous AA-treated N2a cells and DGAT1+2i-treated N2a cells in the context of neuroinvasive virus infection. Taken together, these results reveal that targeting DGAT inhibits neuroinvasive viral infection mainly through inducing ferroptosis.

### Arachidonic acid induces ferroptosis mainly through the ACSL4-LPCAT3-POR pathway to inhibit neuroinvasive virus infection

**Figure 6:**
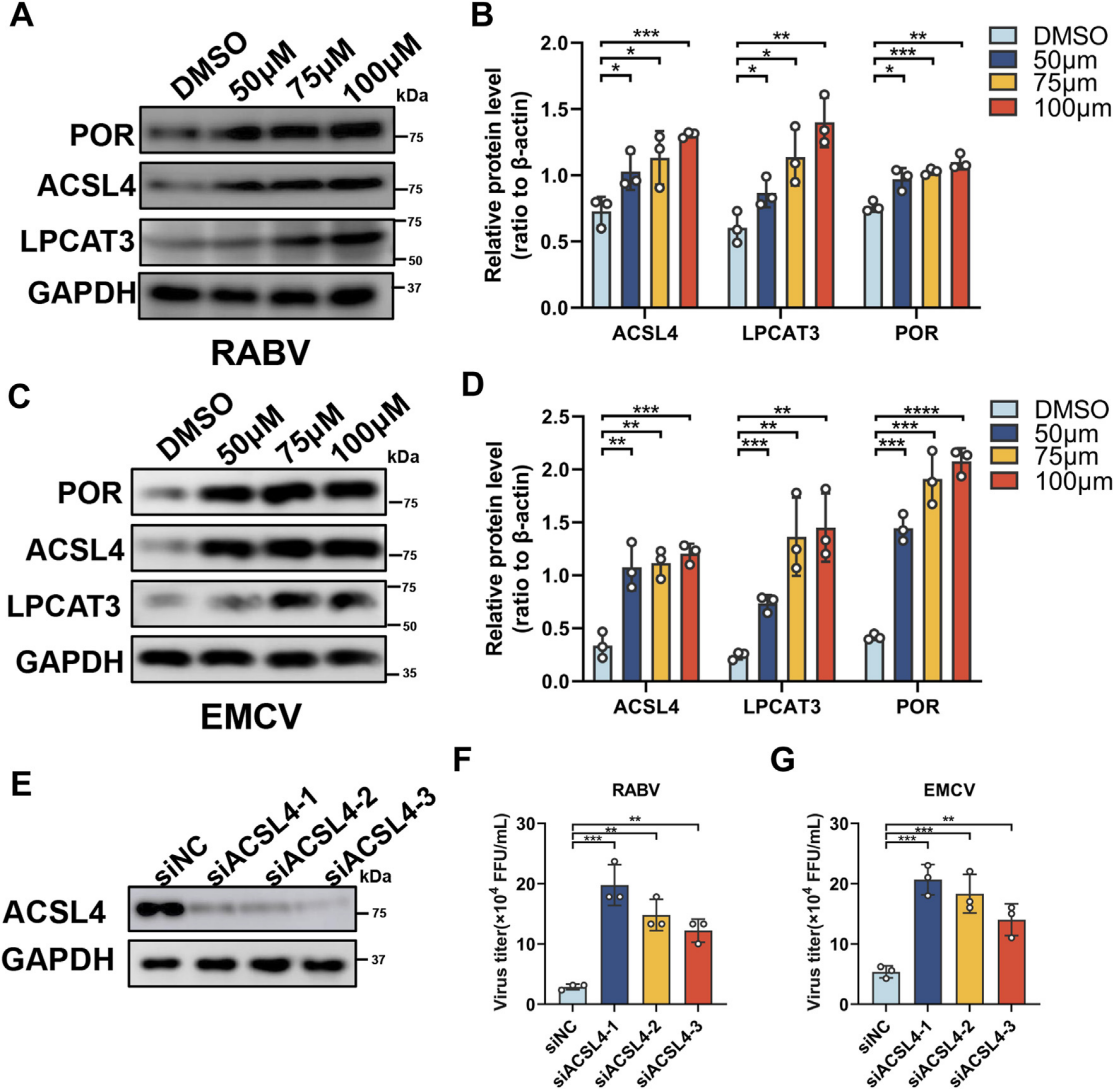
**Arachidonic acid induces ferroptosis through the ACSL4-LPCAT3-POR pathway.** AA-treated N2a cells were infected with RABV (MOI = 0.1) for 24 h; cells were harvested and the expression levels of ACSL4, LPCAT3, and POR determined by Western blotting (*A*) and *gray* values of ACSL4, LPCAT3, and POR were calculated, respectively (n = 3) (*B*). AA-treated N2a cells were infected with EMCV (MOI = 1) for 24 h, cells were harvested and determined ACSL4, LPCAT3 and POR by Western (*C*) and *gray* values of ACSL4, LPCAT3, and POR were calculated, respectively (n = 3) (*D*). N2a cells were transfected with three pairs of small interfering RNA to knock down ASCL4, and the ACSL4 expression was examined by WB at 24 h (*E*). N2a cells transfected with three synthesized siRNAs targeting ACSL4 were then infected with RABV (MOI = 0.1), or EMCV (MOI = 1) for 24 h, and the supernatants were harvested to determine virus titers, respectively (n = 3) (*F* and *G*). Error bars represent mean ± SD. Statistical analysis was determined by Student's *t* test and is notated as follows: ∗*p* < 0.05; ∗∗*p* < 0.01, ∗∗∗*p* < 0.001, and ∗∗∗∗*p* < 0.0001. AA, arachidonic acid; ACSL4, acyl-CoA synthetase long-chain family member 4; EMCV, encephalomyocarditis virus; LPCAT3, lysophosphatidylcholine acyltransferase 3; POR, cytochrome P450 oxidoreductase; RABV, rabies virus; WB, Western blotting.

Acyl-CoA synthetase long-chain family member 4 (ACSL4) plays the critical role in ferroptosis *via* ACSL4-lysophosphatidylcholine acyltransferase 3 (LPCAT3)-cytochrome P450 (POR) pathway, which mainly chooses AA as the key substrate (34). Nevertheless, whether AA would induce ferroptosis through the ACSL4-LPCAT3-POR pathway in the context of neuroinvasive virus infection is unclear. Therefore, to identify this, N2a cells pretreated with different concentrations (50 μM, 75 μM, or 100 μM) of AA were infected with RABV or EMCV, and the infected cells were collected to determine the expression levels of ACSL4, LPCAT3, and POR by Western blotting. Significantly higher levels of ACSL4, LPCAT3, and POR were detected in RABV and EMCV infected N2a cells with AA treatment (Fig. 6, *A* and *C*) and the gray values of each band were also calculated (Fig. 6, *B* and *D*), respectively.

ASCL4 is the key factor in ACSL4-LPCAT3-POR pathway (22, 35), therefore, to investigate whether AA induces ferroptosis mainly through this pathway, three siRNA targeting ACSL4 were synthesized and determined the silencing efficacy (Fig. 6*E*). Furthermore, N2a cells were transfected with siACSL4 and treated with AA (100 μM). Next, the cells were infected with RABV or EMCV, and virus titers were then determined. As shown in Figure 6, *F* and *G*, silencing ACSL4 to restrict the ACSL4-LPCAT3-POR pathway could recover the neuroinvasive virus replication that is inhibited by AA.

**Figure 7:**
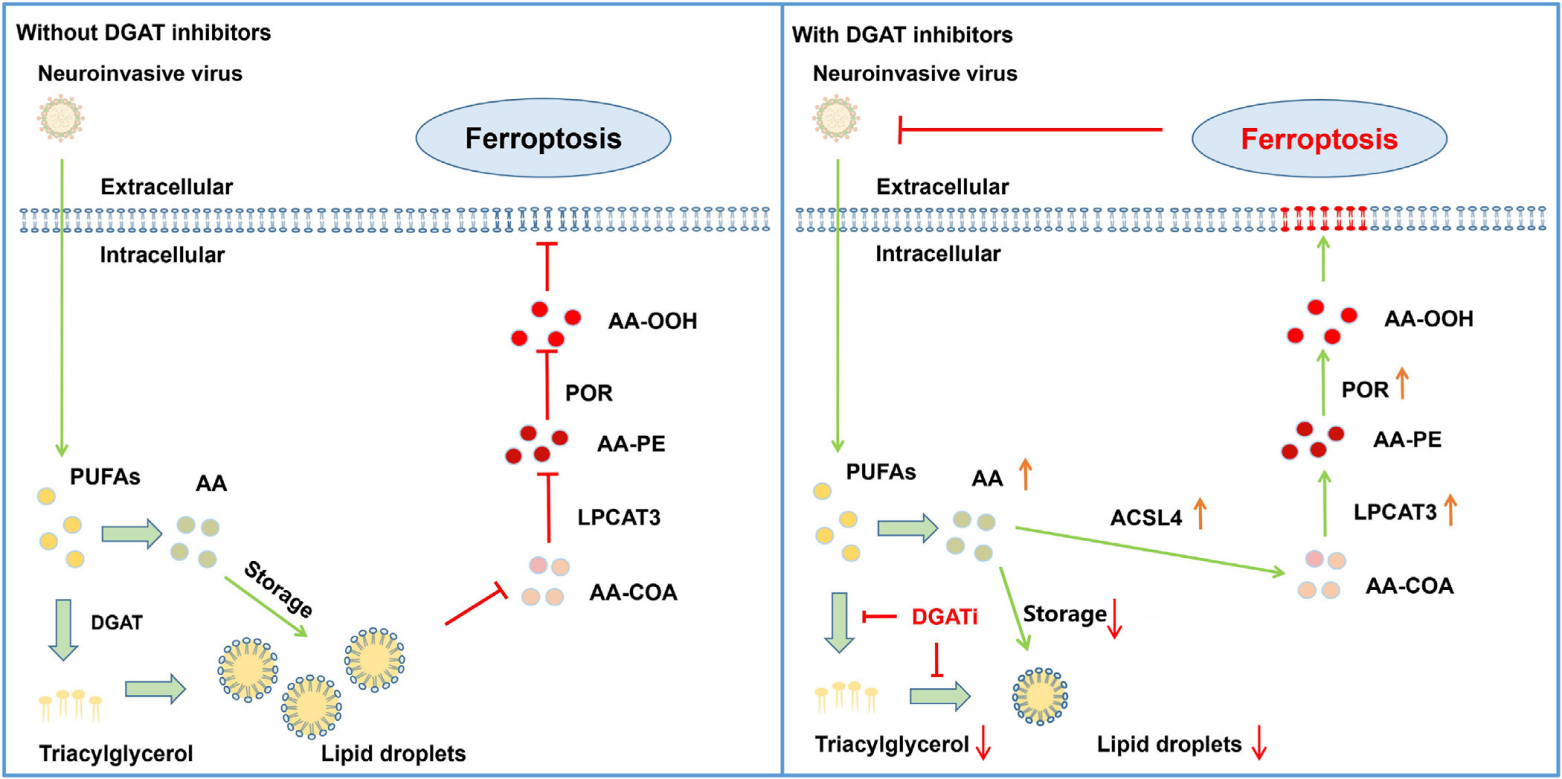
**The schematic diagram of proposed mechanism by which neuroinvasive virus benefits viral replication by restricting arachidonic acid-induced ferroptosis through enhancing LD formation.** Neuroinvasive virus infection induces cellular polyunsaturated fatty acid (PUFAs) biosynthesis through regulating cellular lipid metabolism. Excess PUFAs (including arachidonic acid, AA) can be catalyzed to triacylglycerol (TAG) which further promote LD formation by diacylglycerol acyltransferase 1/2 (DGAT1/2). Reducing LD with DGAT inhibitors can increase the level of cellular AA, which can further induce ferroptosis through activating acyl-CoA synthetase long-chain family member 4 (ACSL4)-lysophosphatidylcholine acyltransferase 3 (LPCAT3)-cytochrome P450 oxidoreductase (POR) pathway to suppress neuroinvasive virus replication. In contrast, without DGAT inhibitors, LD formation can store cellular AA to restrict the ferroptosis, which benefits neuroinvasive virus replication. AA, arachidonic acid; DGAT, diacylglycerol acyltransferase; LD, lipid droplet.

Taken together, these results indicated that AA could induce ferroptosis mainly through the ACSL4-LPCAT3-POR pathway to inhibit neuroinvasive virus replication. Finally, our results indicate that LDs suppressed by DGAT targeting can restrict neuroinvasive virus replication through increasing cellular AA production to induce ferroptosis *via* upregulating ASCL4-LPCAT3-POR pathway, which reveals the protective mechanism of LDs in neuroinvasive virus replication and provides a potential target for drugs development (Fig. 7).

## Discussion

In our study, we revealed that neuroinvasive virus infection could enhance the LD production, and targeting DGAT to inhibit cellular LD formation could result in the release of AA, which could consequently induce ferroptosis to restrict neuroinvasive virus replication. To our knowledge, this is the first report demonstrating the interplay among LDs, ferroptosis and virus infection, providing the specific role of AA in neuroinvasive virus infection. As for a dietary supplement, AA, as an PUFAs, has been demonstrated to sequester TAG to form LDs,which protects cells from lipotoxicity (36, 37). It is beneficial for neuroinvasive virus to form cytosolic AA into LDs, which reduces cytosolic-dependent ferroptosis and maintains cellular morphology for viral replication. Hence, supplying exogenous AA in neuroinvasive virus infection can increase cytosolic AA to induce ferroptosis, assisting the host to clear infected cells and activate immune response to restrict virus replication, which provides a novel viewpoint of antiviral drug development from the metabolic perspective.

Actually, cell death is considered to be a host defense mechanism to inhibit viral replication, and many viruses promote viral replication by evading the death of infected cells. For example, ORF3-protein of porcine epidemic diarrhea virus has been found to promote virus proliferation by inhibiting apoptosis of infected cells (38). For RABV infection, our previous study has demonstrated that G protein–coupled receptor 17 can induce apoptosis to restrict RABV replication, which indicated that cell death after viral infection could restrict RABV replication (39). Additionally, 2A protein of EMCV, another neuroinvasive virus employed in this study, has also been demonstrated to inhibit apoptosis for viral infection and pathogenesis (40). As for another type of PCD, ferroptosis was also found to be restricted by LDs in neuroinvasive virus infection in this study, which expands our understanding the specific mechanisms of viral replication suppressed by various cell death.

Our previous study has demonstrated that RABV infection could induce LD accumulation in N2a cells, and we further found that the infection of other neuroinvasive viruses such as EMCV could also enhance the LD formation in N2a cells and restrict LD formation with DGAT inhibitors could inhibit the viral replication. It is generally believed that increasing LD formation can protect cells from PUFAs-induced lipotoxicity and endoplasmic reticulum stress, indicating that the balance between LD degradation and LD storage will affect the cellular sensitivity of ferroptosis (41, 42). RAB7A-mediated lipophagy increased cellular PUFAs, which enhances RAS-selective lethal 3 (RSL3)-induced ferroptosis in cancer cells. In contrast, tumor protein D52 mediated lipid storage can restrict ferroptosis by forming LDs (43). Fas related factor family member 2 (FAF2), which regulates LD formation and homeostasis, is decreased in the orlistat-mediated ferroptosis in A549 and H1299 cells, indicating that the LD accumulation can inhibit ferroptosis (44). AA is a kind of PUFA that can induce lipid peroxidation to result in ferroptosis, and we found in this study that LDs extracted from RABV infected neuronal cells contains significantly more AA than those collected from uninfected cells (Fig. S3), indicating that LDs are the main storage space for AA, which would prevent AA-induced ferroptosis to facilitate viral replication. In addition, we also observed an enhancement of ferroptosis in mouse brains infected with the neuroinvasive virus; however, the role of ferroptosis in the pathogenicity of neuroinvasive virus infection still needs further investigation.

Lipid peroxidation is one of the main signals of ferroptosis, which is also one of the characteristic signals that distinguish ferroptosis from other PCD (45, 46). Generally, PUFAs can be oxidized by free oxygen or ROS to produce specific oxidized PL, which is different from other forms of PCD (47). In this process, ACSL4 is an essential enzyme involved in lipid metabolism and has also been studied in depth (35). ACSL4 has a substrate preference for AA (48) and then connects AA with CoA to form AA-coenzyme A (AA-CoA), which further forms AA-PE under the catalysis of LPCAT3 (49, 50). AA-PE is prone to peroxidation in enzymatic (arachidonate lipoxygenases [ALOXs] or POR) or nonenzymatic (ROS or Fenton reaction) conditions, which further accumulates on the cell membrane and leads to membrane rupture and ferroptosis eventually (47). In the process of lipid peroxidation, we found that both enzymatic and nonenzymatic (ROS, Fe^2^,^+^ and POR) signals were significantly upregulated in neuroinvasive virus infected cells with AA treatment, indicating that there are many ways to form AA-PE to induce lipid peroxidation. ALOXs have also been reported to play an important role in the oxidation of AA-PE in enzymatic reactions (22). However, based on our previous RNA-sequencing results of RABV infected N2a cells the expression of ALOXs related genes was extremely low, while the expression of POR was significantly high (51). Therefore, we speculated that POR could play a critical role in enzymatic AA-PE catalyzed reaction in neuroinvasive virus infected cells; unfortunately, we did not detect the lipid substrates (AA-CoA, AA-PE, and so on) in the present study, which is warranted in our future study.

ROS production and hydroxyl radical (OH^−^) mediated lipid peroxidation have been reported to lead to plasma membrane damage (52, 53), which is the key event for ferroptosis. Correspondingly, cells have evolved multiple mechanisms to resist this process including antioxidant inhibition or membrane repair. Glutathione peroxidase 4 (GPX4), which belongs to the GPX protein family, is the only GPX member that can convert PL hydroperoxide into PL alcohol thereby effectively limiting ferroptosis (54, 55). Reduced GSH, a cofactor used by GPX4, is a thioalcohol containing tripeptide that is derived from glycine, glutamic acid, and cysteine. Cysteine acts as a speed limiting precursor (56, 57). Solute Carrier Family 7 Member 11 (SLC7A11; also known as xCT) is a transporter subunit in system Xc (58). The SLC7A11-GSH-GPX4 axis is considered to constitute the main cell system to prevent ferroptosis (59, 60). Recent studies have reported many cellular antioxidant mechanisms including the ferroptosis suppressor protein 1 (FSP1)-ubiquinol (CoQH_2_) system (FSP1-CoQH_2_) (61), the dihydroorotate dehydrogenase (DHODH)-CoQH_2_ system (DHODH-CoQH_2_) (62), and the GTP cyclohydrolase 1 (GCH1)-tetrahydrobiopterin (BH_4_) system (GCH1-BH_4_) (63). Consistently, previous studies also indicated that the increase of AA could activate ferroptosis in GPX4 deficient cells, suggesting the critical role of antioxidant systems in AA-dependent ferroptosis (64). However, cellular defense mechanisms of ferroptosis in neuroinvasive virus infection have not been reported yet, and whether these mechanisms are blocked or destroyed within neuroinvasive virus infection is still unclear, which requires further studies to explore.

Taken together, our findings in this study revealed a novel crosstalk among LD, ferroptosis, and neuroinvasive virus infection, providing a potential target of drug development for the neuroinvasive virus.

## Experimental procedures

### Cells, viruses, antibodies, inhibitors, and drugs

N2a (American Type Culture Collection (ATCC) CCL-131), BSR (ATCC CCL-10), BHK-21 (ATCC C-13) cell lines were obtained from ATCC. N2a cells and BSR cells were cultured in Dulbecco's modified Eagle's medium containing 10% fetal bovine serum and 1% penicillin-streptomycin at 37 °C in a humidified 5% CO_2_ atmosphere. Dulbecco's modified Eagle's medium and fetal bovine serum were purchased from Gibco. Penicillin-streptomycin (Cat. No. C0222), DCFH-DA (Cat. No. S0033S), PI (Cat. No. ST511) and Dihydroethidium (Cat. No. S0063) were obtained from Beyotime. An anti-GAPDH antibody (Cat. No. 60004-1-Ig) and an anti-LPCAT3 antibody (Cat. No. 67882-1-Ig) were purchased from Proteintech, an anti-ACSL4 antibody (Cat. No. A20414) and an anti-POR antibody (Cat. No. A5032) were purchased from ABclonal. An anti-4 hydroxynonenal antibody (Cat. No. BS-6313R) was purchased from Bioss. A horseradish peroxidase–conjugated goat anti-mouse antibody (Cat. No. BA1051) and a goat anti-rabbit antibody (Cat. No. BA1055) purchased from Boster, and a DyLight 594 goat anti-mouse IgG (H + L) cross-adsorbed secondary antibody (Cat. No. 35511) and an Alexa Fluor 488 goat anti-mouse IgG (H + L) cross-adsorbed secondary antibody (Cat. No. 11001) were purchased from Invitrogen. The LD probe BODIPY 493/503 (Cat. No. GC42959), N-Oleoyl Leucine (Cat. No. GC44441), PAF C-18:1 (Cat. No. GC41450) and1-Oleoyl Lysophosphatidic Acid (Cat. No. GC42011) were purchased from GlpBio. The DGAT inhibitors A922500 (Cat. No. HY-10038), PF-06424439 (Cat. No. HY-108341A) and Arachidonic acid (Cat. No. HY-109590) were purchased from MedChemExpress. C2-ceramide (Cat. No. A7191), N-Oleoyl Glycine (Cat. No. O9762) and N-palmitoyl glycine (Cat. No. 87081P) were purchased from Sigma-Aldrich. The Triacylglycerol ELISA Kit (Cat. No. BC0620) and Mitochondrial Membrane Potential Assay Kit with JC-1 (Cat. No. M8650) were purchased from Solarbio. Cell Counting Kit-8 (Cat. No. C0005) and ferrostatin-1 (Cat. No. T6500) were purchased from TargetMol. MDA Assay Kit (Cat. No. M496), Liperfluo (Cat. No. L248), FerroOrange (Cat. No. F374) were purchased from DoJindo. The HiScript II 1st Strand complementary DNA (cDNA) Synthesis Kit (Cat. No. R211-01), HiScript III 1st Strand cDNA Synthesis Kit (Cat. No. R222-01) and ChamQ SYBR qPCR Master Mix (Cat. No. Q711-02) were purchased from Vazyme. Arachidonic acid ELISA kit (Cat. No. D751021-0096) was purchased from Sangon Biotech. Lipid Droplet Isolation Kit (Cat. No. ab242290) was purchased from Abcam. TRIzol was purchased from Invitrogen. Prolong Gold antifade mounting solution (Cat. No. P10144) was purchased from Thermo Fisher Scientific.

### Triacylglycerol ELISA

For determining TAG, N2a cells were pretreated with DGAT1i (A922500, 10 μg/ml), DGAT2i (PF06424439, 60 μg/ml) or DGAT1+2i (A922500+PF06424439) and infected with RABV (MOI = 0.1, 24 h) or EMCV (MOI = 1, 24 h). Cells were harvested to test cellular triacylglycerol by ELISA according to the manufacturer’s instructions. The absorbance values were read by a SpectraMax 190 spectrophotometer (Molecular Devices), and the triacylglycerol level was calculated based on the standard curve.

For determining AA, N2a cells were pretreated with DGAT1i (A922500, 10 μg/ml), DGAT2i (PF06424439, 60 μg/ml), DGAT1+2i (A922500+PF06424439) or AA(100 μM), infected with RABV (MOI = 0.1 or 1) or EMCV (MOI = 1) for 24 h. Cells were harvested and determined AA concentration by arachidonic acid ELISA kit. For determining AA concentration in cytosol and LDs, the LDs from infected N2a cells were then isolated by Lipid Droplet Isolation Kit (Abcam), and the AA concentration in the cell lysates were determined by the ELISA kit to indicate the cytosol AA level.

### Confocal microscopy

Coverslips pretreated with polylysine were cultured with N2a cells which were pretreated with DGAT1i (A922500, 10 μg/ml), DGAT2i (PF06424439, 60 μg/ml), or DGAT1+2i (A922500+PF06424439) and infected with RABV (MOI = 0.1, 24 h) or EMCV (MOI = 1, 24 h), and determined as previously described (9). Cells were treated with 4% paraformaldehyde at room temperature for 30 min, followed by 15 min of incubation in PBS solution containing 0.1 M glycine, washed with PBS and permeabilized with 0.1% Triton-X100 in PBS at room temperature for 30 min, then blocking solution (10 mM Tris–HCl pH 7.5, 150 mM NaCl, 2% bovine serum albumin, and 10% goat serum) was added for 30 min at room temperature. Washed with PBS, cells were stained with BODIPY 493/503 (room temperature for 30 min) and 4′,6-diamidino-2-phenylindole (room temperature for 5 min), respectively. Coverslips were incubated with Prolong Gold antifade mounting solution to prevent quenching and imaged with a confocal microscope (Zeiss) with 488, 561, and 405 nm laser lines for excitation of Alexa Fluor 488, Alexa Fluor 594, and 4′,6-diamidino-2-phenylindole, respectively.

### Virus titration

For RABV determination, viral titers were tested by using a direct fluorescence assay as previously described (9, 65). Supernatants were diluted 10-fold into plates in quadruplicate, and BSR cells were added to wells and incubated at 37 °C for 48 h. Plates were fixed with 80% cold acetone for 2 h at −20 °C, and incubated with FITC-RABV-P antibody for 1 h at 37 °C. A fluorescence microscope (ZEISS) was used to count the positive foci and virus titers were calculated and expressed as focus-forming units per ml (FFU/ml).

For EMCV determination, supernatants of cells infected with EMCV were diluted 10-fold into plates in quadruplicate, and BHK-21 cells were added to wells and incubated at 37 °C for 48 h. Cytopathic effect was observed and virus titers were calculated and expressed as plaque forming units per ml (PFU/ml).

### Western blotting

Harvested cells were washed with cold PBS and lysed with radio-immunoprecipitation assay buffer. After centrifuging with 12,000*g* at 4 °C, supernatants were collected and added SDS loading buffer for boiling to form samples. Samples were added into SDS-PAGE gels and transferred to polyvinylidene fluoride membranes (Merck Millipore). After blocking with 5% skimmed milk for 1 h, membranes were incubated with the indicated primary antibody at 4 °C overnight. Washed with Tris buffered saline with Tween-20 for three times, membranes were incubated with the indicated secondary antibody for 1 h at room temperature. Signals were displayed by an Amersham Imager 600 (GE HealthCare) imaging system.

### RT-qPCR analysis

Total cells were collected, extracted by TRIzol and reverse transcribed with a HiScript III 1st Strand cDNA Synthesis Kit. Primers for testing viral genome RNA were designed: vRNA-F, ACAGGCAACACCACTGATAA; vRNA-R, TCAGCGGGACATATTCAGGA, which were added into reaction volumes with the following program (Bio-rad): 95 °C for 2 min for one cycle followed by 40 cycles at 95 °C for 5 s and 60 °C for 30 s.

### Lipidomics analysis

N2a cells pretreated with DMSO or DGATi (A922500 10 μg/ml + PF06424439 60 μg/ml) and infected with RABV (MOI = 0.1, 24 h). Cells were harvested and methanol (0.75 ml) was added to samples, which were placed into a glass tube with a Teflon lined cap, and the tube was vortexed. Subsequently, 2.5 ml of MTBE was added and the mixture was incubated for 1 h at room temperature in a shaker. Phase separation was induced by adding 0.625 ml of MS-grade water. Upon 10 min of incubation at room temperature, the sample was centrifuged at 1000*g* for 10 min. The upper (organic) phase was collected, and the lower phase was reextracted with 1 ml of the solvent mixture (MTBE/methanol/water (10:3:2.5, v/v/v)), and collecting the upper phase. Combined organic phases were dried and dissolved in 100 μl of isopropanol for storage. Then analyzed by LC-MS/MS. UHPLC-MS/MS analyses were performed using a Vanquish UHPLC system (Thermo Fisher Scientific) coupled with an Orbitrap Q ExactiveTM HF mass spectrometer (Thermo Fisher Scientific) in Novogene Co, Ltd. The raw data files generated by UHPLC-MS/MS were processed using the Compound Discoverer 3.01 (CD3.1, Thermo Fisher Scientific, https://mycompounddiscoverer.com/2019/08/23/compound-discoverer-3-1-released/) to perform peak alignment, peak picking, and quantitation for each metabolite. The normalized data were used to predict the molecular formula based on additive ions, molecular ion peaks, and fragment ions. And then peaks were matched with the LIPID MAPS and LipidBlast database to obtain accurate qualitative and relative quantitative results.

### The median CC_50_ assay

Cytotoxicity of identified lipid substances on N2a cells were determined by cell counting kit 8 as previously described (66). N2a cells were cultured in 96-well plates, treated with gradient concentrations of lipid substances at 37 °C for 48 h. Cell culture was replaced with fresh medium containing 10% cell counting kit 8 solution and incubated at 37 °C for 45 min. The absorbance was measured at 450 nm *via* a SpectraMax 190 spectrophotometer (Molecular Devices). Series of Concentration of lipid substances were compared with DMSO-treated cells respectively in GraphPad Prism 8.0 (GraphPad Software , https://www.graphpad.com/features) by a sigmoidal nonlinear regression to fit dose-response curves, and every CC_50_ of lipid substances was calculated individually depending on the specific curve.

### Cell death assay

N2a cells were infected with RABV and determined cell death *via* PI as previously described (67, 68). Erastin (10 μm) was used as a positive control. Cells were harvested, washed with cold PBS, and stained with PI for 30 min at 37 °C. After washing with PBS, cells were counted by a cell counter (Countstar). A total of 10^4^ cells were extracted to determine the fluorescence intensity *via* a fluorescent microplate reader (Tecan).

### Lipid peroxidation, Fe^2+^, ROS, and 4-HNE assay

Lipid peroxidation was tested by Liperfluo (green), and Fe^2+^ concentration was tested by FerroOrange according to the manufacturer’s instructions (Dojindo). Cellular ROS level was tested by DCFH-DA according to the manufacturer’s instructions (Beyotime). 4-HNE was tested by 4-HNE antibody according to the manufacturer’s instructions (Bioss). N2a cells were with AA (100 μm) and infected with RABV (MOI = 0.1, 24 h) or EMCV (MOI = 1, 24 h). Erastin (10 μm) was used as a positive control. Cells were harvested, washed with cold PBS, and stained with Liperfluo (Ex: 488 nm, Em: 550 nm), FerroOrange (Ex: 543 nm, Em: 580 nm) or DCFH-DA (Ex: 488 nm, Em: 525 nm) for 30 min at 37 °C. Cells were stained with 4-HNE antibody for 60 min and stained with Alexa Fluor 488 goat anti-mouse IgG (H + L) for 45 min at 37 °C. After washing with PBS, cells were counted by a cell counter (Countstar). Subsequently, 10^4^ cells were extracted to determine the fluorescence intensity *via* a fluorescent microplate reader (Tecan). For ROS determination, cells were also tested *via* flow cytometry (Beckman Coulter).

### MDA assay

Cellular MDA concentration was measured by MDA Assay Kit (Dojindo). N2a cells were treated with AA (100 μm) and infected with RABV (MOI = 0.1, 24 h) or EMCV (MOI = 1, 24 h). Erastin (10 μm) was used as a positive control. Cells were harvested and washed with cold PBS. A small part of cell suspensions was lysed and determined the total protein level. The other part of the cell suspensions was lysed according to the manufacturer’s instructions (Dojindo). The thiobarbituric acid included in the kit was added to the supernatants of cell homogenate to form a thiobarbituric acid-MDA mixture, which was determined *via* a fluorescent microplate reader (TECAN) (Ex: 540 nm, Em: 590 nm).

### siRNA transfection

Three types of siRNA used in this study were designed and synthesized by Tsingke Biotechnology. To verify the transfection efficiency, siRNAs were diluted to a final concentration of 50 nM and transfected into N2a cells according to the manufacturer’s instructions. The specific target sequence for siACSL4-1 was 5′ - CGCUAUGGCAAAGAGAAUA - 3′, and siACSL4-2 sequence was 5′ - GCAGAGAUAUC AUGCUUUA - 3′, and siACSL4-3 sequence was 5′ - CACACCGAUUCAUGAAUGU - 3′.

### Statistical analysis

All data were analyzed by using GraphPad Prism 8 (GraphPad Software, CA). The mean fluorescence intensity was calculated by ImageJ software (https://imagej.nih.gov/ij/). Data are representative of three independent experiments, with a representative experiment being shown. For all the results, an unpaired two-tailed *t* test or one-way ANOVA was used to determine whether differences were statistically significant. The following notations are used to indicate significant differences between groups: ∗*p* < 0.05, ∗∗*p* < 0.01; ∗∗∗*p* < 0.001; and ∗∗∗∗*p* < 0.0001.

## Data availability

All lipidomics data used in this study have been uploaded to the EMBL-EBI MetaboLights database and identified MTBLS9363 (Fig. 2) and MTBLS9386 (Fig. S2), which can be accessed at https://www.ebi.ac.uk/metabolights/MTBLS9363 and https://www.ebi.ac.uk/metabolights/MTBLS9386.

## Supporting information

This article contains supporting information.

## Conflict of interest

The authors declare that they have no conflicts of interest with the contents of this article.
