# Supplementary material for: Neuroinvasive virus facilitates viral replication by employing lipid droplets to reduce arachidonic acid-induced ferroptosis
Source: J Biol Chem. 2024 Mar 13;300(4):107168. doi: 10.1016/j.jbc.2024.107168 (PMC10999822; doi:10.1016/j.jbc.2024.107168)
Supplement: Supporting Information [file mmc1.docx]

Supplementary Figures for

Neuroinvasive virus facilitates viral replication by employing lipid droplets to reduce arachidonic acid-induced ferroptosis

Jianqing Zhao^1,3,4#^, Qianruo Wang^5,#^, Zhenkun Liu^1,3,4^, Mai Zhang^1,3,4^, Jinquan Li^5^, Zhen F Fu^1,3,4^, Ling Zhao^1,2,3,4^*, Ming Zhou^1,3,4^*

^1^National Key Laboratory of Agricultural Microbiology, Huazhong Agricultural University, Wuhan 430070, China.

^2^Hubei Hongshan Laboratory, Wuhan 430070, China.

^3^Key Laboratory of Preventive Veterinary Medicine of Hubei Province, College of Veterinary Medicine, Huazhong Agricultural University, Wuhan 430070, China.

^4^Frontiers Science Center for Animal Breeding and Sustainable Production, Wuhan 430070, China.

^5^College of Biomedicine and Health, College of Life Science and Technology, Huazhong Agricultural University Wuhan 430070, China.

^#^Equal contribution

*Corresponding Authors:

Ming Zhou, mikchail@163.com; mingzhou@mail.hzau.edu.cn.

Ling Zhao, zling604@outlook.com; lingzhao@mail.hzau.edu.cn.

**
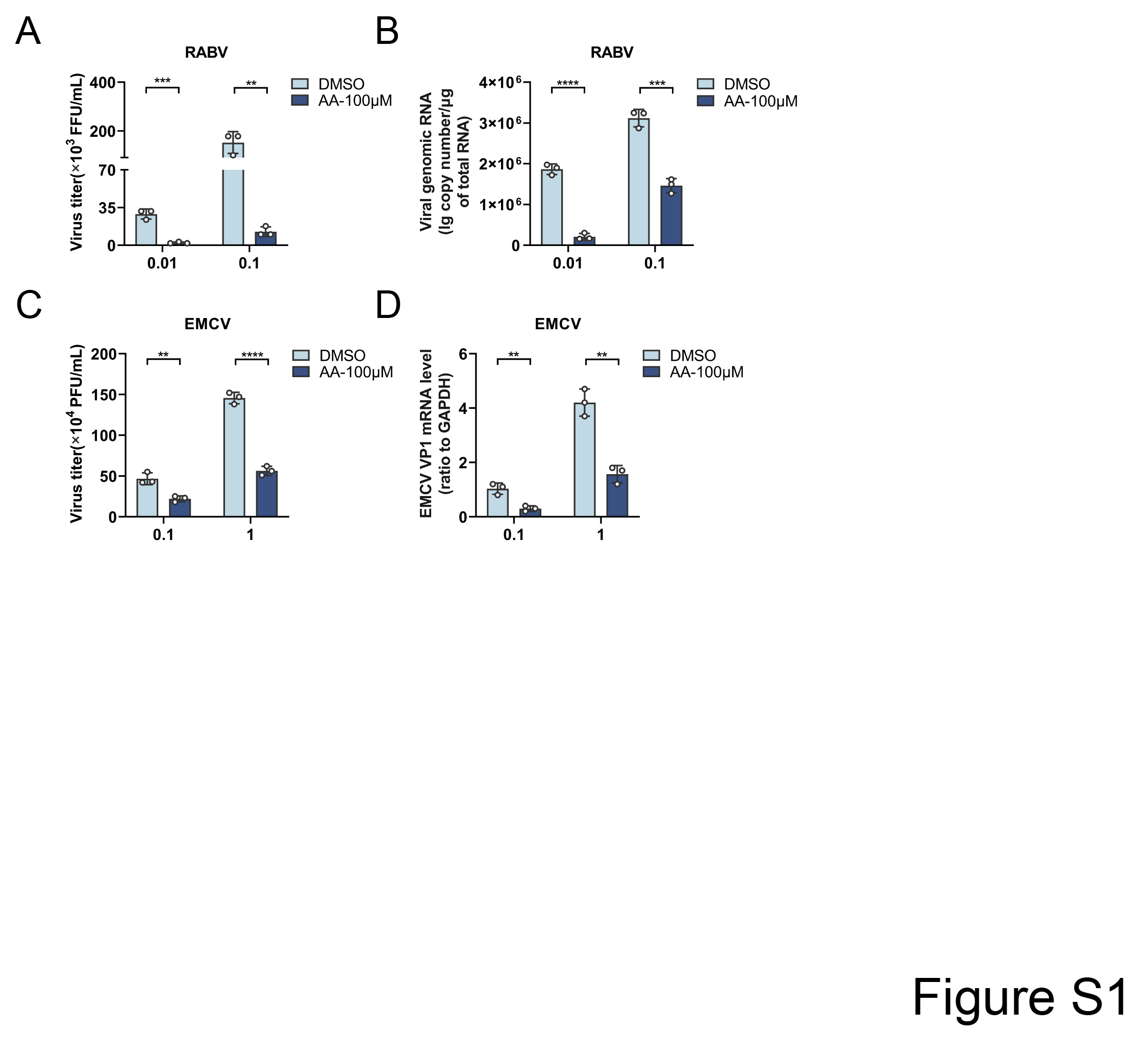
**

**Fig. S1** Arachidonic Acid restricts neurotropic virus production.

N2a cells were treated with Arachidonic acid (100 μM) and infected with RABV (MOI=0.01 or 0.1) for 24 h, supernatants were harvested and determined virus titers (n=3) (A) and viral genome RNA was tested by qPCR (n=3) (B). N2a cells were pretreated with AA in several concentration (100 μM) and infected with EMCV (MOI=0.1 or 1) at 24 h, supernatants were harvested to determine virus titers (n=3) (C) and cells were collected to test VP1 mRNA level via qPCR (n=3) (D). Statistical analysis was determined by one-way ANOVA or Student's t test and notated as follows: *, P<0.05; **, P<0.01; ***, P<0.001 and ns, no significant.


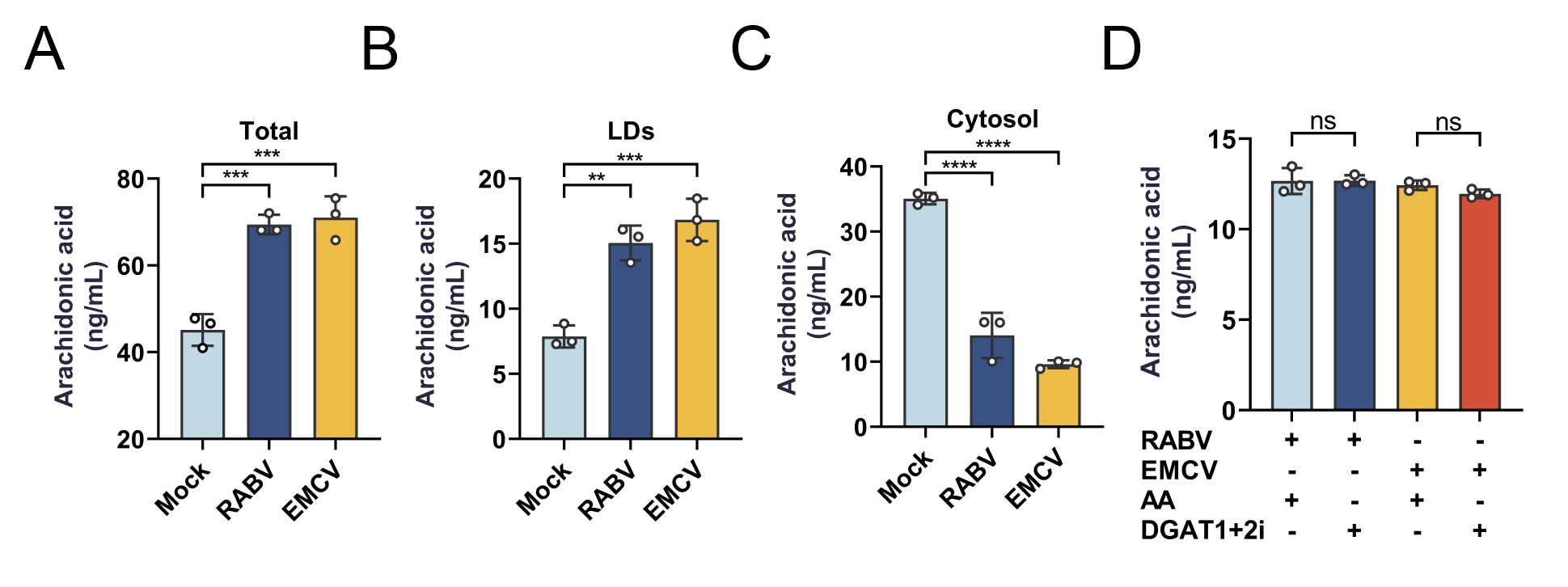


**Fig. S2 The specific cellular concentrations of AA in N2a cells**.

N2a cells were infected with RABV (MOI=1), EMCV (MOI=1) or mock infected for 24 h. Cellular total AA concentrations (A), AA concentrations in LDs (B) and cytosolic AA concentrations (C) were determined by ELISA (n=3), respectively. N2a cells were treated with AA (100 μM) or DGAT1+2i (A922500, 10 μg/ml + PF06424439, 60 μg/ml), and infected with RABV (MOI=0.1), EMCV (MOI=1) for 24 h, Cellular AA concentrations (D) were determined by ELISA (n=3). Statistical analysis was determined by one-way ANOVA and notated as follows: **, P<0.01; ***, P<0.001 and ns, no significant.

**
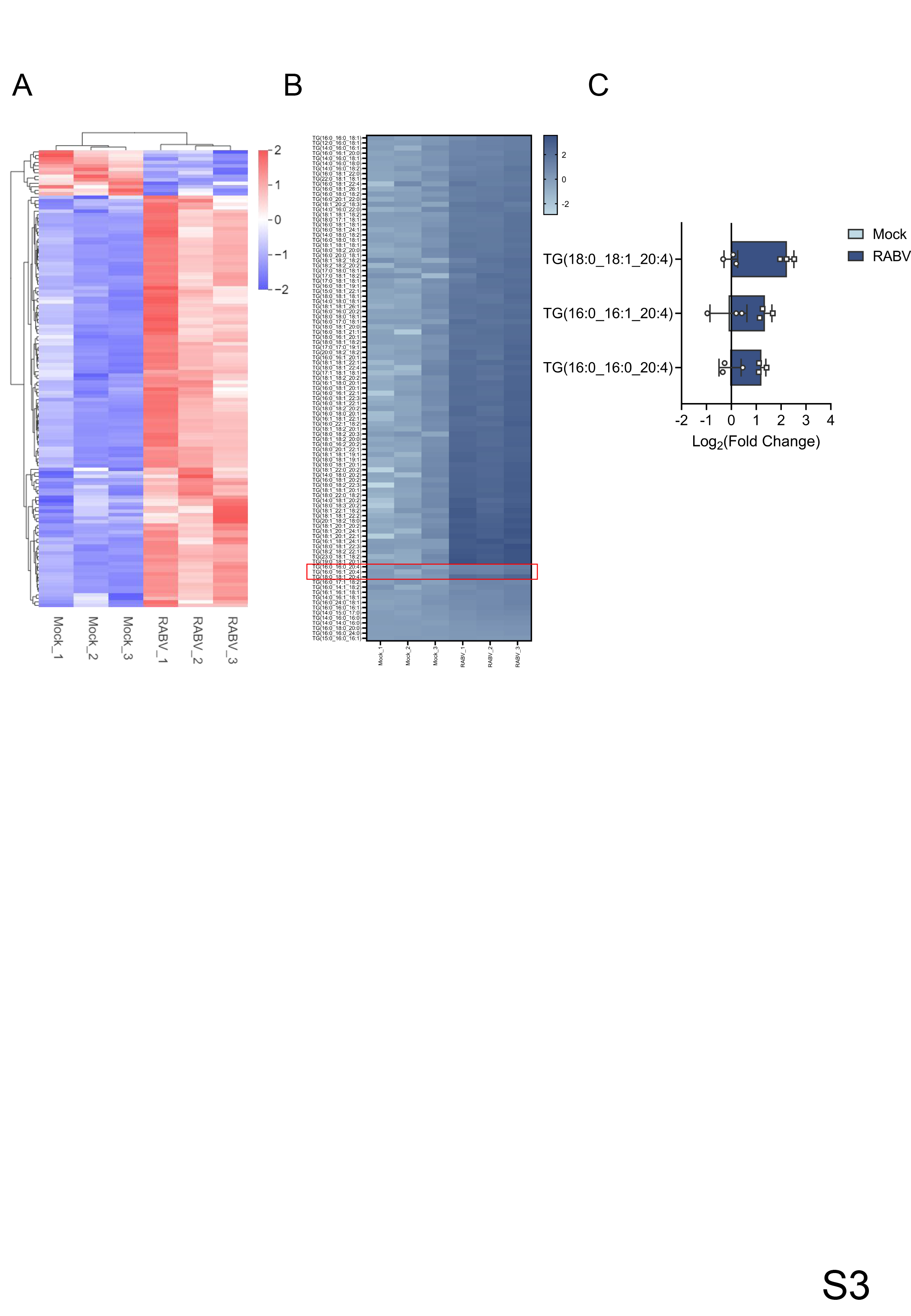
**

**Fig. S3 Non-targeted lipidomics analysis of lipid droplets in RABV-infected N2a cells.**

N2a cells were infected with RABV (MOI=1) or mock infected with DMSO for 24 h. The cells were then collected, and lipid droplets were extracted with a commercial kit. The extracted lipid droplets were then subjected to lipidomics analysis. (A) Heatmap of differential levels of fatty acids within the extracted LD. Each row is corrected for the Z value. Longitudinal is the clustering of samples, and horizontal is the clustering of fatty acid. (B) The changes of TG content within the extracted LD. (C) The changes of arachidonic acid levels including TG (18:0_18:1_20:4), TG (16:0_16:1_20:4), and TG (16:0_16:0_20:4).


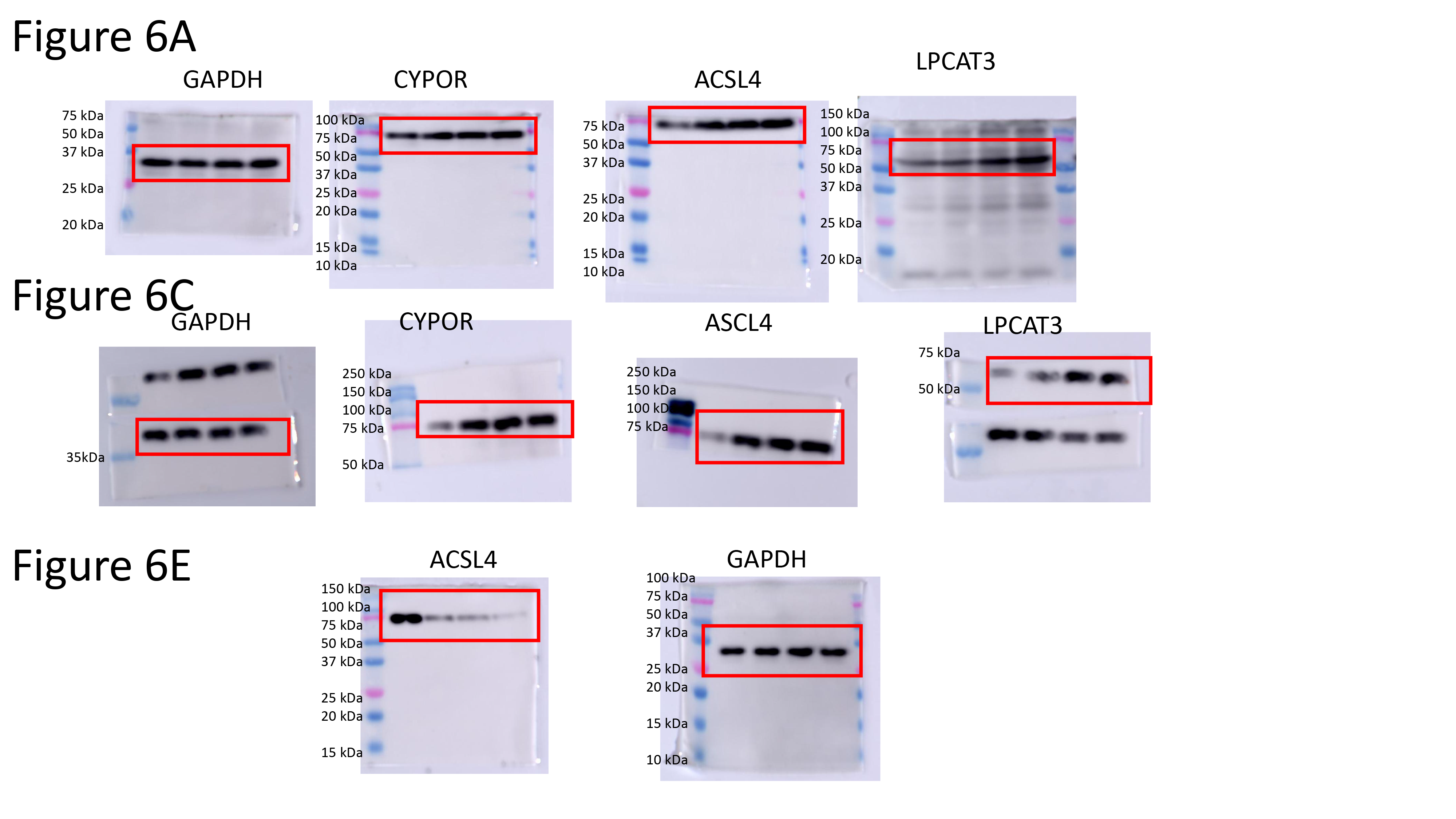


**Fig. S4 The uncropped western blots used in Figure 6**
